# Supplementary material for: Estimating recent migration and population-size surfaces
Source: PLoS Genet. 2019 Jan 14;15(1):e1007908. doi: 10.1371/journal.pgen.1007908 (PMC6347299; doi:10.1371/journal.pgen.1007908)
Supplement: S1 Appendix — (PDF) [file pgen.1007908.s001.pdf]

1 S1 Appendix

1.1 The model

The coalescent process for two samples under a multi-deme model can be described by a continuous time Markov chain (CTMC) (Bahlo and Griffiths, 2001). Let i, j represent sampled lineages and α, β their locations, respectively, d is the number of demes (or populations) and $(\alpha, \beta) \in \{1, \cdots, d\} \times \{1, ..., d\}$. Let c denote the coalescent state. The infinitesimal rate matrix R of this CTMC is

$$\begin{aligned}
R_{(\alpha,\beta),(\gamma,\beta)} &= m_{\alpha,\gamma} \;\; \beta = 1, ..., d, \; \gamma \neq \alpha \\
R_{(\alpha,\beta),(\alpha,\gamma)} &= m_{\beta,\gamma} \;\; \alpha = 1, ..., d, \; \gamma \neq \beta \\
R_{(\alpha,\alpha),(c)} &= q_\alpha \\
R_{(\alpha,\beta),(\alpha,\beta)} &= -(m_{\alpha_+} + m_{\beta_+}) - \delta_{\alpha\beta} q_\alpha \\
R_{(c),(c)} &= 0 \\
R_{(c),\beta} &= 0 \;\; \beta = 1, ..., d \\
R_{(\alpha,\beta),(\gamma,\kappa)} &= 0 \;\; \gamma, \, \kappa = 1, ..., d, \; \gamma \neq \alpha, \; \kappa \neq \beta,
\end{aligned} \tag{S1}$$

where $M = \langle m_{\alpha,\beta} \rangle$ denotes the migration rate matrix, and $m_{\alpha,\beta}$ is the migration rate between demes α, β and $q_\alpha = \frac{1}{2N_\alpha}$ is the coalescent rate of deme α which is proportional to the inverse of the population size at deme α (N_k), and $m_{\alpha_+} = \sum_{\gamma \neq \alpha} m_{\alpha,\gamma}$. Let $T_{i,j}$ denote the (random) coalescent time between the pair of sampled lineages, and $f_{T_{i,j}}(t)$ denote the probability density of a coalescent event at time t. Here, we derive $f_{T_{i,j}}(t)$ by conditioning on the position of the two lineages.

Lemma 1.1 *Let* $\big(X_i(t), X_j(t)\big) \in \{1, \cdots, d\} \times \{1, ..., d\}$ *denote the position of lineage i and lineage j at time t respectively and are moving according to the CTMC defined by (S1). The probability density $f_{T_{i,j}}(t)$ that lineage i and j coalesce at time t is given by* $\sum_{\kappa=1}^{d} q_\kappa P(X_i(t) = \kappa, X_j(t) = \kappa)$.

For $\Delta t \approx 0$,

$$P(T_{i,j} \in [t, t + \Delta t]) \tag{S2}$$

$$\approx \sum_{\kappa=1}^{d} P(T_{i,j} \in [t, t+\Delta t] | X_i(t) = \kappa, X_j(t) = \kappa) P(X_i(t) = \kappa, X_j(t) = \kappa) \tag{S3}$$

$$\approx \sum_{\kappa=1}^{d} q_\kappa \Delta t P(X_i(t) = \kappa, X_j(t) = \kappa). \tag{S4}$$

Taking the limit $\Delta t \to 0$, we arrive at the density

$$f_{T_{i,j}}(t) = \lim_{\Delta t \to 0} \ P(T_{i,j} \in [t, t + \Delta t])/\Delta t = \sum_{\kappa=1}^{d} q_\kappa P(X_i(t) = \kappa, X_j(t) = \kappa). \tag{S5}$$

The random walk approximation to the coalescent

Here, we introduce an approximation,

$$P(X_i(t) = \kappa, X_j(t) = \kappa) \approx P(X_i(t) = \kappa) P(X_j(t) = \kappa). \tag{S6}$$

The intuition is that the probability that lineage i and j coalesce before time t is extremely small such that the two lineages approximately behave like two independently moving particles. Each lineage can be modeled by a random walk with transition matrix M. These assumptions were also made in the context of continuous spatial diffusion models for haplotype sharing Baharian et al. (2016); Ringbauer et al. (2017) , and even further back, as a general approximation to the two-dimensional continuous-space coalescent process (Barton et al., 2002; Wilkins, 2004; Blum et al., 2004; Novembre and Slatkin, 2009; Robledo-Arnuncio and Rousset, 2010).

This approximation implies that

$$f_{T_{i,j}}(t) \approx \sum_{\kappa} q_k (e^{-Mt})_{\alpha,\kappa} (e^{-Mt})_{\beta,\kappa}, \tag{S7}$$

where lineages i, j are initially sampled in deme α, β. Or equivalently in matrix form,

$$f_{T_{i,j}}(t) \approx \left(e^{-Mt} Q e^{-Mt} \right)_{i,j}, \tag{S8}$$

where $Q = diag(q_1, ..., q_d)$.

Varying migration rates and population sizes across time

Corollary 1.1.1 *Let time slice k be defined by the interval $t_{k-1} < t < t_k$, M_k denote the migration rate matrix in time slice k, and $Q_k = diag(q_1^k, ..., q_d^k)$ where q_α^k denotes the coalescent rate in deme α at time slice k. Let $T_{i,j}$ denote the coalescent time between lineage i, j sampled in demes α, β, then under the independence assumption, for $t \in (t_{K-1}, t_K)$,*

$$f_{T_{i,j}}(t) \approx \Big(G_K(t) Q_K G_K(t)\Big)_{\alpha,\beta}, \tag{S9}$$

where $G_K(t) = \Big(\prod_{k=1}^{K-1} \exp\big(-(t_k - t_{k-1})M_k\big)\Big) \exp\big(-(t - t_K)M_K\big)$

Expected number of lPSC segments given the demography Θ

Lemma 1.2 *It follows that $E[X_{i,j}^\mu|\Theta] \approx G \int_\mu^\infty f_L(l|\Theta)/l\, dl$ where $X_{i,j}^\mu$ denotes the number of PSC segment greater than μ basepairs shared between haploid individuals i, j, Θ denotes the demographic model, G denote the size of the genome, L denotes the random length (in base-pairs) of the PSC segment between i and j containing a pre-specified position in the genome, and $f_L(l|\Theta)$ the probability densite of L conditional on Θ.*

Let $E[\mathcal{F}^\mu|\Theta]$ denote the expected fraction of the genome between i, j that lies in PSC segments greater than μ, and $E[s^\mu|\Theta]$ the expected size of a PSC segment conditional on it being at least length μ. According to equations 9-14 from (Palamara et al., 2012),

$$E[X_{i,j}^\mu|\theta] \approx \frac{G\, E[\mathcal{F}^\mu|\Theta]}{E[s^\mu|\Theta]}, \tag{S10}$$

$$E[\mathcal{F}^\mu|\Theta] = \int_\mu^\infty f_L(l|\Theta) dl, \tag{S11}$$

$$E[s^\mu|\Theta] = \frac{\int_\mu^\infty f_L(l|\Theta) dl}{\int_\mu^\infty f_L(l|\Theta)/l\, dl}. \tag{S12}$$

We obtain the desired result by substituting (S11) and (S12) into (S10) and canceling like-terms.

Expected age of a segment

We choose PSC segment lengths based on their expected age which is derived below.

Lemma 1.3 *The expected coalescent time (t, in generations) of PSC segments between length L_1 centiMorgans and L_2 centiMorgans is approximately $\frac{300}{4}(\frac{1}{L_1}+\frac{1}{L_2})$ if the effective population size (N) is sufficiently large.*

We choose to work in units of basepairs, and will convert back to units of morgans at the end. We convert L_1 into units of base-pairs with the transformation: $\mu = \frac{L_1}{100r}$ and similarly $\nu = \frac{L_2}{100r}$.

Let us denote $T|l, N$ as the random coalescent time of a PSC segment that is at least length l under a single-deme demography model with population size N. The expected coalescent time of an PSC segment longer than μ base-pairs can be expressed as

$$\begin{aligned} E[T|l \geq \mu, N] = \int_0^\infty t f_T(t|l \geq \mu, N)dt = \int_0^\infty t\frac{f_L(l \geq \mu|t)f_T(t|N)}{f_L(l \geq \mu|N)}dt \\ = \frac{\int_0^\infty t f_L(l \geq \mu|t)f_T(t|N)dt}{\int_0^\infty f_L(l \geq \mu|t)f_T(t|N)dt}, \end{aligned} \tag{S13}$$

where $f_L(l|t) = 4r^2t^2le^{-2trl}$ denotes the probability density that a PSC segment is of length l given it has a common ancestor event at time t, $f_T(t|N)$ denotes the probability density that a coalescent event occurs at time t under the demography model with population size N.

Next, we expand a key term in equation (S13)

$$f_L(l \geq \mu|t) = \int_\mu^\infty f_L(l|t)dl = (2rt\mu + 1)\exp\left(-2rt\mu\right) \tag{S14}$$

and assume,

$$f_T(t|N) = \frac{e^{-t/N}}{N}. \tag{S15}$$

Putting everything together,

$$E[T|l \geq \mu, N] = \frac{N(1+6Nr\mu)/(1+2Nr\mu)^3}{(1+4Nr\mu)/(1+2Nr\mu)^2} = \frac{N(1+6Nr\mu)}{1+6Nr\mu+8N^2(r\mu)^2}. \tag{S16}$$

We can remove the dependence of N by taking $\lim_{N\to\infty}$ as done similarly in Baharian et al. (2016),

$$\lim_{N\to\infty} E[T|l \geq \mu, N] = \frac{3}{4r\mu} \tag{S17}$$

Now that we have derived the expected age of PSC segment longer than μ, it is quite simple

to expand the equation for PSC segments between μ and ν base-pairs,

$$E[T|\mu \leq l \leq \nu] = \frac{\int_0^\infty t f_L(\mu \leq l \leq \nu|t) f_{T|N}(t)dt}{\int_0^\infty f_L(\mu \leq l \leq \nu|t) f_{T|N}(t)dt} = \frac{\int_0^\infty t\Big(f_L(l \geq \mu|t) - f_L(l \geq \nu|t)\Big) f_{T|N}(t)dt}{\int_0^\infty \Big(f_L(l \geq \mu|t) - f_L(l \geq \nu|t)\Big) f_{T|N}(t)dt}$$
$$= \frac{3}{4}\left(\frac{1}{r\mu} + \frac{1}{r\nu}\right) \tag{S18}$$

We transform back to units of centimorgans: let $L_1 = 100r\mu$ and $L_2 = 100r\nu$ be in units of centiMograns, we get the desired result

$$\lim_{N\to\infty} E[T|\mu \leq l \leq \nu] = 75\left(\frac{1}{L_1} + \frac{1}{L_2}\right). \tag{S19}$$

1.2 Transformation of migration rates to dispersal rates

Migration rates inferred under a discrete model can be transformed to dispersal distances representing parameters in continuous space. Here, we derive the transformation.

Lemma 1.4 *Consider a random walk on a 2D grid, where steps are taken according to a Poisson process with rate m measured in generations, and let $\underline{X}(t)$ be a vector denoting the coordinates of the particle at time t. The distribution of $\underline{X}(t)$ approximately only depends on the compound parameter $m(\Delta x)^2$ (or equivalently $\sqrt{m}\Delta x$) where Δx denotes the step size in the grid (i.e. edge length).*

$$\underline{X}(t) = \sum_{i=1}^{N(t)} \underline{Z}_i, \tag{S20}$$

where $N(t)$ is the number of steps taken by time t, and $\underline{Z}_i$ is a random variable representing the direction and magnitude taken at step i. Since $X(t)$ is a sum of iid variables, a form of the central limit theorem applies here and $X(t)$ converges to the normal distribution (Rényi, 1960).

In a random walk on a triangular grid, a particle can move in one of the 6 directions (upper-right, right, lower-right, left, upper-left, and lower-left):

$$
\begin{aligned}
&\underline{Z}_i \\
&\quad = (\Delta x/2, \Delta x\sqrt{3}/2)^T \text{ with } p = 1/6 \\
&\quad = (\Delta x, 0)^T \text{ with } p = 1/6 \\
&\quad = (\Delta x/2, -\Delta x\sqrt{3}/2)^T \text{ with } p = 1/6 \\
&\quad = (-\Delta x, 0)^T \text{ with } p = 1/6 \\
&\quad = (-\Delta x/2, \Delta x\sqrt{3}/2)^T \text{ with } p = 1/6 \\
&\quad = (-\Delta x/2, -\Delta x\sqrt{3}/2)^T \text{ with } p = 1/6
\end{aligned}
$$

where Δx represents the step size in the grid (i.e. edge length). The mean and variance are given by,

$$E[\underline{X}(t)] = 0 \tag{S21}$$

and,

$$Var[\underline{X}(t)] = \frac{mt(\Delta x)^2}{2} I_2. \tag{S22}$$

where I_2 is the identity matrix. We do not show the additional steps of using the law of total variance conditioning on $N(t)$ and using $E[N(t)] = mt$. Under normality, the mean and variance are sufficient statistics. Note that (S21) and (S22) also hold for square grids.

Interpretation of the migration diffusion parameter $m(\Delta x)^2$

Here, we discuss how we interpret the diffusion constant $m(\Delta x)^2$. Let the distance $d = \|\underline{X}(t)\| = \sqrt{x_1^2 + x_2^2}$, then

$$E[d^2]/t = E[x_1^2 + x_2^2]/t = E[x_1^2]/t + E[x_2^2]/t = \frac{m(\Delta x)^2}{2} + \frac{m(\Delta x)^2}{2} = m(\Delta x)^2. \tag{S23}$$

Thus the square root of the diffusion constant can be written as $\sqrt{m}\Delta x = \sqrt{\frac{E[d^2]}{t}}$ which suggests an interpretation as the distance traveled by an individual *after one generation*, and sometimes is called the "dispersal" distance or the "root mean square dispersal distance". In these units, dispersal is more directly comparable to empirical estimates of dispersal from pedigree data Kaplanis et al. (2018). However, interpolating the distance traveled over many generations is not trivial here because the the distance does not scale linearly with time (i.e. the units of dispersal distance are km per square root generation).

1.3 Diversity rates versus coalescent rates

For computational efficiency, the EEMS software uses a combination of the resistance distance model and within-deme "diversity rates" to approximate expected pairwise coalescent times, in which,

$$E[\hat{T}_{\alpha,\beta}] = \begin{cases} \frac{R_{\alpha,\beta}}{4} + \frac{e_{q_\alpha}+e_{q_\beta}}{2} & \text{if } \alpha \neq \beta \\ e_{q_\alpha} & \text{if } \alpha = \beta \end{cases}. \tag{S24}$$

where $E[\hat{T}_{\alpha,\beta}]$ is the resistance distance approximation to the expected coalescent time between deme α and deme β, e_{q_α} is the "diversity rate" in deme α, and $R_{\alpha,\beta}$ is the resistance distance between demes α, β (Petkova et al., 2016). The diversity rates have no simple expression in terms of population-genetic parameters under the multi-deme coalescent model. As an alternative, diversity rates can be interpreted as reflecting average within deme heterozygosity since $e_q = E[\hat{T}_w] \propto H_\alpha$ where the heterozygosity for deme α (H_α) is defined as,

$$H_\alpha = \frac{1}{\binom{n_\alpha}{2}} \sum_{i<j, i\in\alpha, j\in\alpha} D_{i,j}, \tag{S25}$$

where $D_{i,j}$ is the average number of differences between (haploid) individuals i and j.

Migration and population sizes are identifiable in MAPS

MAPS models the recombination process using rates estimated from a recombination rate map. In this model, population sizes and migration rates can be inferred separately rather than as a joint parameter. Intuitively, the recombination rate serves an independent clock to calibrate estimates.

More formally, a statement of identifiability is a statement regarding the likelihood. MAPS models the expected number of lPSC segments shared between pairs of (haploid) individuals, and can be computed with an integral. The integral can be broken up into a product of two functions: a function describing the decay of PSC segments as a function of time ("recombination rate clock"), and the coalescent time probability density $f_{T_{i,j}}(t)$. The migration rates and population sizes only appear in $f_{T_{i,j}}(t)$, and cannot be factored into parameters involving combinations of the migration rates and population sizes.

1.4 The prior

The structure of the prior closely resembles the prior in the EEMS method Petkova et al. (2016). The tessellation for the migration rates (T_m) is encoded by a list ($\underline{l^m}, \underline{m}, c_m, \mu_m$)

where $\underline{l^m}$ are the locations of each cell, $\underline{m}$ the rates of each cell, and are vectors of length c_m (i.e. number of Voronoi cells), and μ_m is the overall mean migration rate. The Voronoi tessellation for the coalescent rates is $T_q = (\underline{l^q}, \underline{q}, c_q, \mu_q)$.

The location of each (unordered) Voronoi cell is distributed uniformly across the habitat,

$$l_c^m \overset{iid}{\sim} U(H), \tag{S26}$$

where U denotes the uniform distribution. The number of cells (a-priori) are drawn from a negative binomial distribution,

$$c_m \sim \text{NegBi}(r_m, p_m). \tag{S27}$$

The effects of each Voronoi cell is normally distributed with variance ω^2.

$$\log 10(m_i) \overset{iid}{\sim} N(\mu_m, \omega_m^2) \tag{S28}$$

$$\log 10(q_i) \overset{iid}{\sim} N(\mu_q, \omega_q^2) \tag{S29}$$

The probability of a particular (unordered) cell configuration is,

$$p(\underline{m}|c_m) = c_m! \prod_{i=1}^{c_m} N(\log 10(m_i)|\mu_m, \omega_m^2) \tag{S30}$$

We assume,

$$\log 10(\omega_m) \sim U(-3, \log 10(1.5)) \tag{S31}$$

$$\log 10(\omega_q) \sim U(-3, log10(1)) \tag{S32}$$

We set 1.5 as the upper bound for $\log 10(\omega_m)$. For the normal distribution, 95% of the density is within two standard deviations of the mean. As a result, by setting the upper bound for for $\log 10(\omega_m)$ to be 1.5, we are constraining m so that the probability that it is within 3 orders of magnitude from the mean is 0.95 *a priori*, and similarly we set 1 as the upper bound for $\log 10(\omega_q)$ to restrict the population sizes so to be within 2 orders of magnitude from the mean with probability 0.95 *a priori*.

We assume,

$$\mu_m \sim U(-10, 4) \tag{S33}$$

$$\mu_q \sim U(-10, 4). \tag{S34}$$

We place a uniform prior on the log of the mean rates to reflect that we are uncertain about the order of magnitude. Here, the data is highly informative of the mean, as a result, we can allow the support of the prior to vary by many orders of magnitude.

1.5 MCMC

Re-parameterization

In this section, we describe how we try to decorrelate parameters in order to improve mixing of the MCMC. We decouple the migration rates from the mean rate (μ), and variance (ω) by introducing a new variable e_i,

$$e_i \overset{iid}{\sim} N(0, 1). \tag{S35}$$

We can write the cell specific migration rates as,

$$\log10(m_i) = e_i\omega + \mu. \tag{S36}$$

Written this way, we can make separate updates to the cell effects (e_i), the mean (μ) and the variance scale (ω), instead of for example, updating m_i which depends on all three parameters.

Furthermore, we want to ensure that the mean cell effect is zero, i.e. $\bar{e} = \frac{\sum_i e_i}{c} \approx 0$ and have the parameter μ to absorb any change in the mean cell effects. This does not always happen in practice because of poor MCMC mixing. To ensure that the mean cell effect is zero, we add MH joint random-walk updates to μ and e_i as follows,

$$\mu' = \mu + \epsilon, \tag{S37}$$

$$e_i' = e_i - \frac{\epsilon}{\omega}, \tag{S38}$$

where $\epsilon \sim N(0, 1)$. The intuition here is that a constant ϵ is subtracted from the mean, which then gets added to the individual cell effects e_i which ensures that the mean cell effect is approximately 0.

The steps above are applied to both the migration rates and coalescent rates.

Updating the number of cells

The number of cells change the dimension of the likelihood, and a result, we must use a Reversible Jump MCMC step so that the ratio of densities in the Metropolis-Hastings acceptance ratio is well-defined (Green, 1995). We choose to update the number of cells with a birth-death update (Stephens, 2000). Fortunately, in such a case, the updates reduce to standard Metropolis-Hastings because the dimension matching constant (i.e. the "Jacobian") equals one (Petkova et al., 2016; Stephens, 2000). See equations S31 and S32 in Petkova et al. (2016) for formulas regarding the birth-death update. Here, we use nearly identical updates (with a slight modification).

When increasing the number of cells from c to $c+1$ (i.e. a birth-update), we randomly choose a location uniformly across the habitat, and the new migration is proposed from a standard normal because our cell effects are standardized to improve MCMC mixing as discussed above. In contrast, EEMS proposes cell effects migration to be normally distributed around a cell effect at a randomly chosen point in the habitat. Here we set, p(birth) = p(death) = 0.5 if the number cells ≥ 1, otherwise p(birth) = 1.

The acceptance ratio for a birth update (going from c cells to $c+1$ cells) is

$$\alpha(x, x') = min(1, \frac{p(\text{death})}{p(\text{birth})} \frac{l(x')p(x')\frac{1}{c+1}}{l(x)p(x)N(e_{c+1}|0,1)}), \tag{S39}$$

where x denotes the current state of the MCMC, x' the proposed state, e_{c+1} is the proposed cell effect drawn from a standard normal, $l()$ is the likelihood function, and $p()$ is the prior. Conversely, in a death-update, we randomly choose one cell uniformly to kill. In this case, the acceptance ratio for a death proposal (going from $c+1$ cells to c cells) is

$$\alpha(x, x') = min(1, \frac{p(\text{birth})}{p(\text{death})} \frac{l(x')p(x')N(e_c|0,1)}{l(x)p(x)\frac{1}{c+1}}). \tag{S40}$$

References

Soheil Baharian, Maxime Barakatt, Christopher R Gignoux, Suyash Shringarpure, Jacob Errington, William J Blot, Carlos D Bustamante, Eimear E Kenny, Scott M Williams, Melinda C Aldrich, et al. The Great Migration and African-American Genomic Diversity. *PLoS Genetics*, 12(5):e1006059, 2016.

Melanie Bahlo and Robert C Griffiths. Coalescence Time for Two Genes from a Subdivided Population. *Journal of Mathematical Biology*, 43(5):397–410, 2001.

Nick H Barton, Frantz Depaulis, and Alison M Etheridge. Neutral Evolution in Spatially Continuous Populations. *Theoretical Population Biology*, 61(1):31–48, 2002.

Michael GB Blum, Christophe Damerval, Stephanie Manel, and Olivier François. Brownian Models and Coalescent Structures. *Theoretical Population Biology*, 65(3):249–261, 2004.

Peter J Green. Reversible Jump Markov Chain Monte Carlo Computation and Bayesian Model Determination. *Biometrika*, 82(4):711–732, 1995.

Joanna Kaplanis, Assaf Gordon, Tal Shor, Omer Weissbrod, Dan Geiger, Mary Wahl, Michael Gershovits, Barak Markus, Mona Sheikh, Melissa Gymrek, et al. Quantitative analysis of population-scale family trees with millions of relatives. *Science*, (early online): eaam9309, 2018.

John Novembre and Montgomery Slatkin. Likelihood-Based Inference in Isolation-by-Distance Models Using the Spatial Distribution of Low-Frequency Alleles. *Evolution*, 63(11):2914–2925, 2009.

Pier Francesco Palamara, Todd Lencz, Ariel Darvasi, and Itsik Peer. Length Distributions of Identity by Descent Reveal Fine-scale Demographic History. *The American Journal of Human Genetics*, 91(5):809–822, 2012.

Desislava Petkova, John Novembre, and Matthew Stephens. Visualizing Spatial Population Structure with Estimated Effective Migration Surfaces. *Nat Genet*, 48(1):94–100, Jan 2016. doi: 10.1038/ng.3464.

A Rényi. On the Central Limit Theorem for the Sum of a Random Number of Independent Random Variables. *Acta Mathematica Hungarica*, 11(1-2):97–102, 1960.

Harald Ringbauer, Graham Coop, and Nicholas H Barton. Inferring Recent Demography from Isolation-By-Distance of Long Shared Sequence Blocks. *Genetics*, 205(3):1335–1351, 2017.

JJ Robledo-Arnuncio and Rousset. Isolation by Distance in a Continuous Population under Stochastic Demographic Fluctuations. *Journal of Evolutionary Biology*, 23(1):53–71, 2010.

Matthew Stephens. Bayesian Analysis of Mixture Models with an Unknown Number of Components, an Alternative to Reversible Jump Methods. *Annals of Statistics*, pages 40–74, 2000.

Jon F Wilkins. A Separation-of-Timescales Approach to the Coalescent in a Continuous Population. *Genetics*, 168(4):2227–2244, 2004.
