# Supplementary figures and images for: Estimating recent migration and population-size surfaces
Source: PLoS Genet. 2019 Jan 14;15(1):e1007908. doi: 10.1371/journal.pgen.1007908 (PMC6347299; doi:10.1371/journal.pgen.1007908)

(a) Simulation

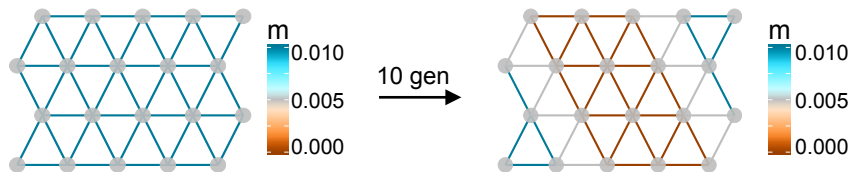

(b) Inference

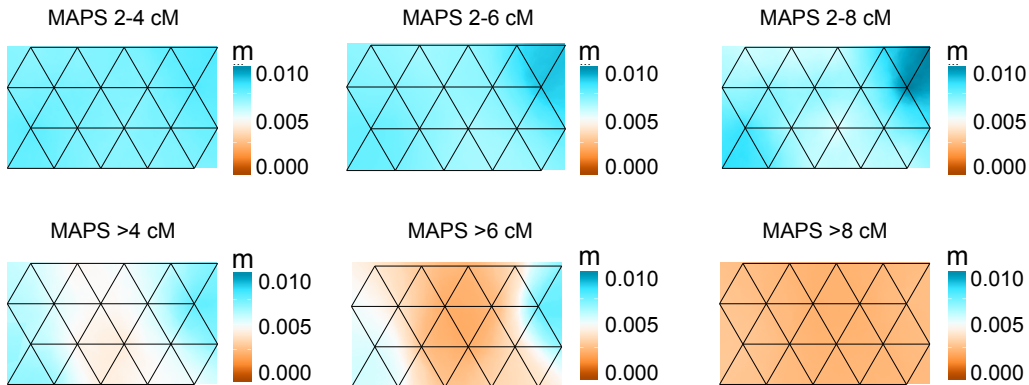

Supplement: S1 Fig — Here, we investigate the ability of MAPS to detect a recent barrier (< 10 generations) for various PSC length bins (a) Simulation scenario. Population sizes were set to 10,000 per deme and 10 diploids were sampled per deme, replicating the conditions in Fig 2b. (b) Results for different PSC length bins. Length bins that encompass shorter segments (2-4cM 2-6cM 2-8cM) recover the higher uniform migration surface; while length bins with longer segments (>4, >6, >8) recover the recent ancestral barrier. For the last length scale (> 8cM), the signature of low migration extends across the habitat. The variation in migration rates is missed presumably because of the small number of shared segments at this length scale. (PDF) [file pgen.1007908.s002.pdf]

## (a) Simulation

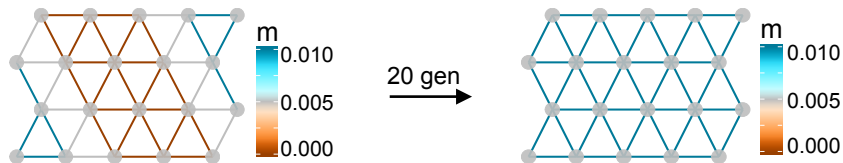

## (b) Inference

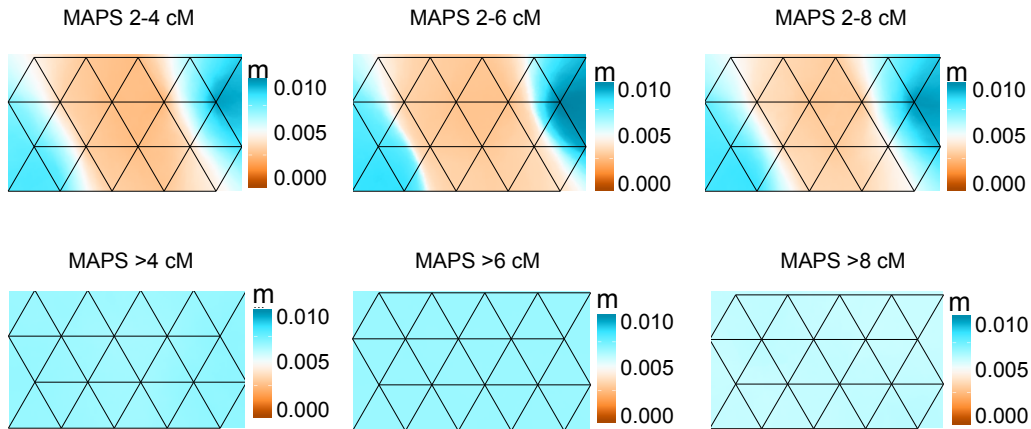

Supplement: S2 Fig — a) Simulation scenario. Population sizes were set to 10000 per deme and 10 diploids were sampled per deme, replicating the conditions in Fig 2c. (b) Results for different PSC length bins. Length bins that encompass shorter segments (2-4cM, 2-6cM, 2-8cM) recover the ancestral barrier; while length bins with longer segments (>4, >6, >8) recover the recent constant migration surface. (PDF) [file pgen.1007908.s003.pdf]

Simulation

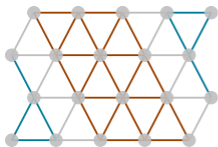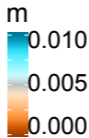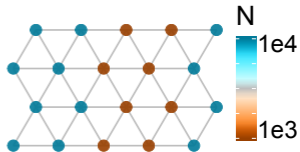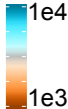

MAPS &gt; 2 cm

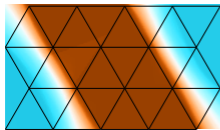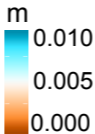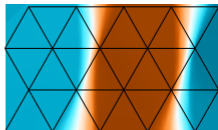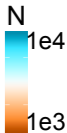

Supplement: S3 Fig — a) Simulation Scenario. Heterogeneous population-sizes and migration rates (as shown) were simulated, and 10 diploid individuals were sampled per deme. (b) Results for PSC segments greater than 2cM are shown. (PDF) [file pgen.1007908.s004.pdf]

a)

1-5cM (~2700 years)

effective size

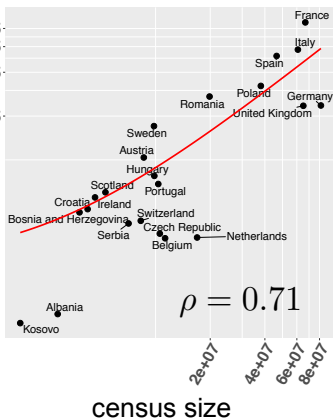

b)

5-10cM (~675 years)

effective size

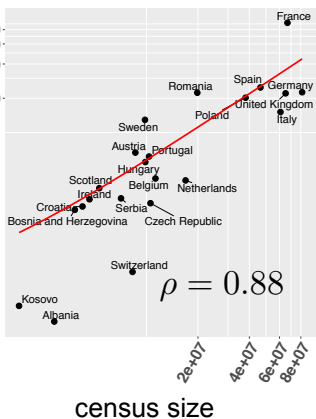

c)

&gt;10cM (~225 years)

effective size

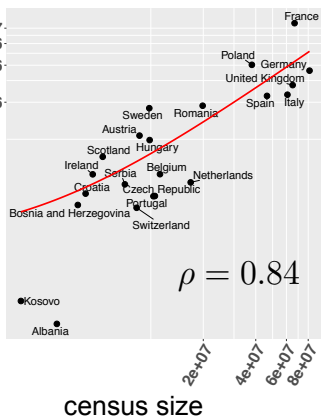

Supplement: S5 Fig — Using the MAPS output, we estimate a total size per population by summing the estimated deme-level sizes across the area of each respective country (whether’s a deme’s location falls within a country was determined by querying [46]). Finally, we plot the results on a log10 scale for different length scales (a) 1-5cM, (b) 5-10cM, and (c) >10cM. The red curve denotes the linear fit on the absolute scale. Note Kosovo and Albania as candidate outliers possibility because of cryptic relatedness artificially decreasing population sizes. (PDF) [file pgen.1007908.s006.pdf]

a)

5-10cM vs 1-5cM

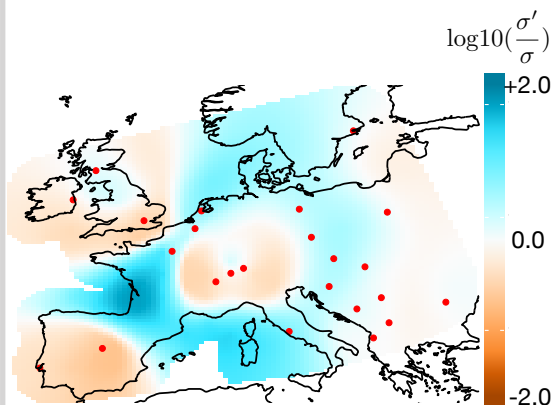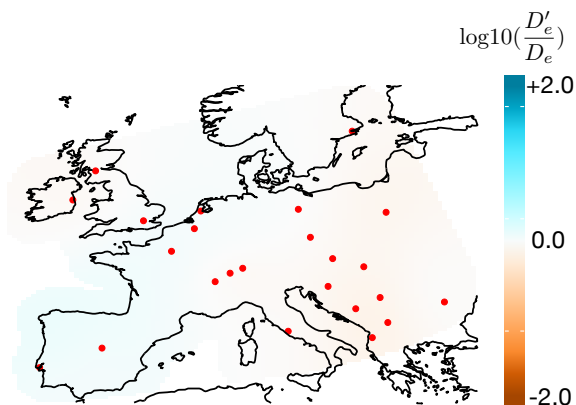

b)

&gt;10cM vs 5-10cM

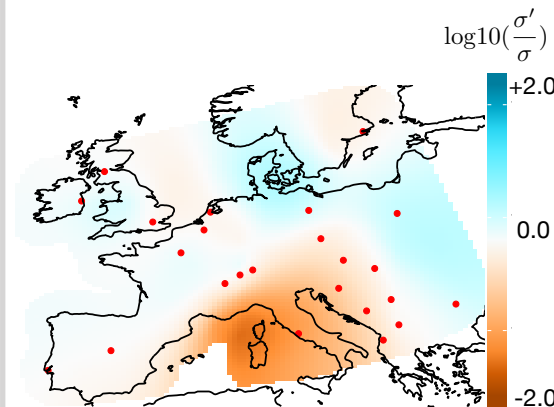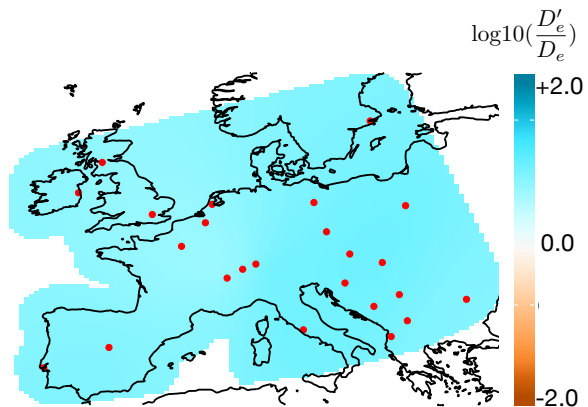

Supplement: S6 Fig — (a) We plot estimates of E[log10(σ′σ)] and E[log10(De′De)] across the spatial habitat where σ′ (De′) denotes the dispersal rates (population densities) in the 5-10cM length bin and σ (De) denotes the dispersal rates (population densities) in the 1-5cM length bin. (b) The results here are similarly plotted as above, however, the adjacent length scales are given by: 5-10cM and >10cM. The log10 differences are estimated in such a way so that the mean log10 difference is shrunk to zero. For example, for estimating dispersal in 5-10cM, we assume log10(σ′) = E[log10(σ)] + ϵ where E[log10(σ)] is estimated using PSC segments 1-5cM and ϵ ∼ N(0, ω2) is estimated from PSC segments 5-10cM. Consequently, the log ratio between dispersal rates from the two lengths bins is constructed to have mean zero apriori (i.e. E[log10(σ′σ)]=0). (PDF) [file pgen.1007908.s007.pdf]

# EEMS

(a)

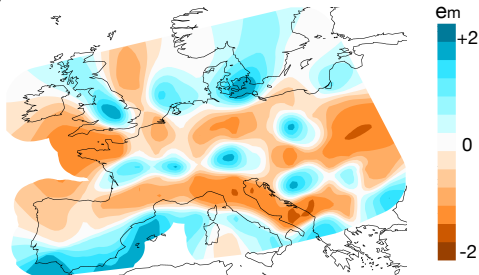

(b)

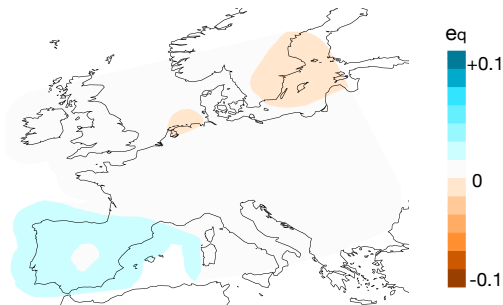

Supplement: S7 Fig — We apply EEMS to the same set of individuals as used in Fig 4 (see Methods). (a) The effective migration rates (b) The effective diversity rates. Here, we ran EEMS with 200 demes (as in Fig 4) with default parameters and averaged over 10 independent replicate chains. Each chain ran with 50e6 MCMC iterations, 25e6 set as burn-in, and we thinned every 5000 iterations. (PDF) [file pgen.1007908.s008.pdf]

(a)

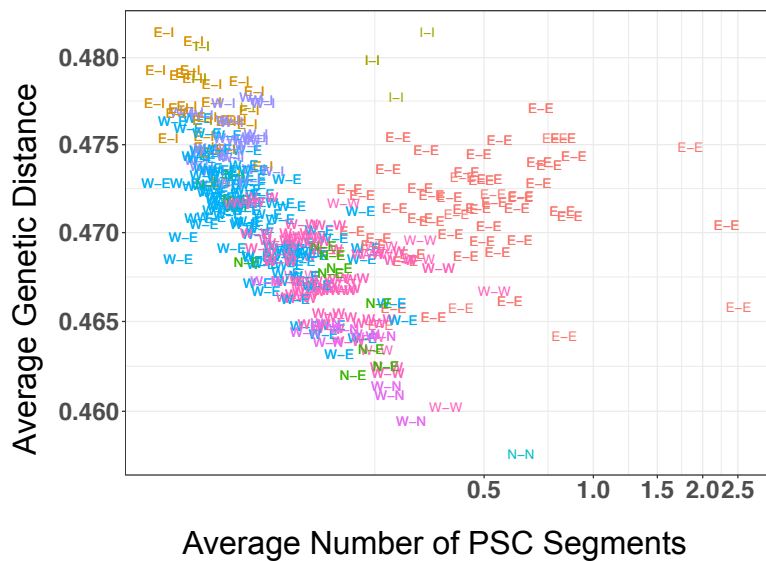

(b)

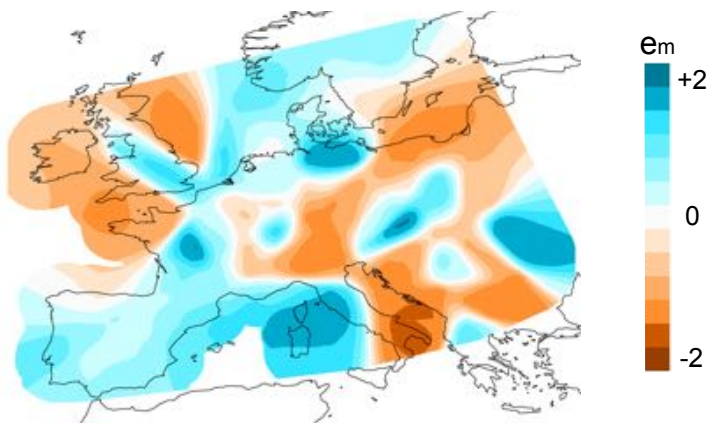

Supplement: S8 Fig — (a) The averaged genetic distance (as used in EEMS) is plotted against the average number of PSC segments (>1cM) for each pair of populations. Each point denotes a pair, the symbols represent groupings from [20] (W Western Europe, S Southern Europe, I Italian & Iberian Peninsula, and E Eastern Europe), and the colors represent the pair of regions. We see a negative correlation between the two summary statistics (Pearson’s ρ = -0.38, p-value = 7e-11), with the largest deviations occurring in comparisons between Eastern European populations. (b) EEMS results on PSC data transformed to a distance matrix. First, we encoded the PSC sharing statistics into a similarity matrix S such that Si,j is the number of shared PSC segments between samples i and j and Si,i is the maximum number of shared segments in the dataset (which we denote as c) to ensure S is a similarity matrix. Next, we transformed S to a genetic distance matrix D such that D = c11T − S + E where E ≈ 0 is a random genetic distance matrix of normal vectors with mean 0 and standard deviation of 0.01 added to ensure D is full rank. Finally, we applied EEMS to the distance matrix D. Though this procedure is heuristic, we see shared features between this surface and the MAPS dispersal surface shown in Fig 4. (PDF) [file pgen.1007908.s009.pdf]
