# Supplementary material for: Estimating recent migration and population-size surfaces
Source: PLoS Genet. 2019 Jan 14;15(1):e1007908. doi: 10.1371/journal.pgen.1007908 (PMC6347299; doi:10.1371/journal.pgen.1007908)
Supplement: S4 Fig — The color scheme is the same as used in [20] where the colors give categories based on the regional groupings: W Western Europe, S Southern Europe, and E Eastern Europe (a) The average sharing within each sample locale is transformed to an estimate of effective population size using an equation in Appendix B of [19]. The equation can be roughly summarized as to say that Nα∝1x¯α,α where Nα is the effective population size in deme α and x¯α,α is the average pairwise PSC sharing between individuals in deme α. (b) Similar to [20], for each focal population (marked with an x), we plot the normalized average pairwise sharing between that population and all others (normalized by the average sharing within the focal population), i.e. if α is the focal population, we show x¯α,βx¯α,α for each other country β. (PDF) [file pgen.1007908.s005.pdf]

a)

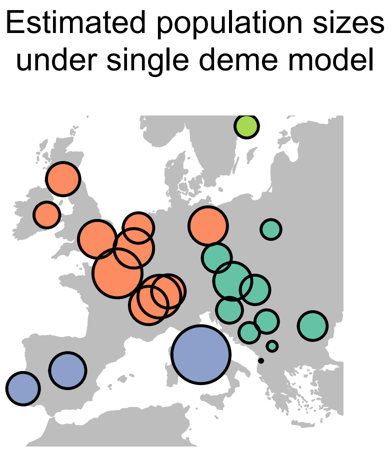
Estimated population sizes
under single deme model

b)

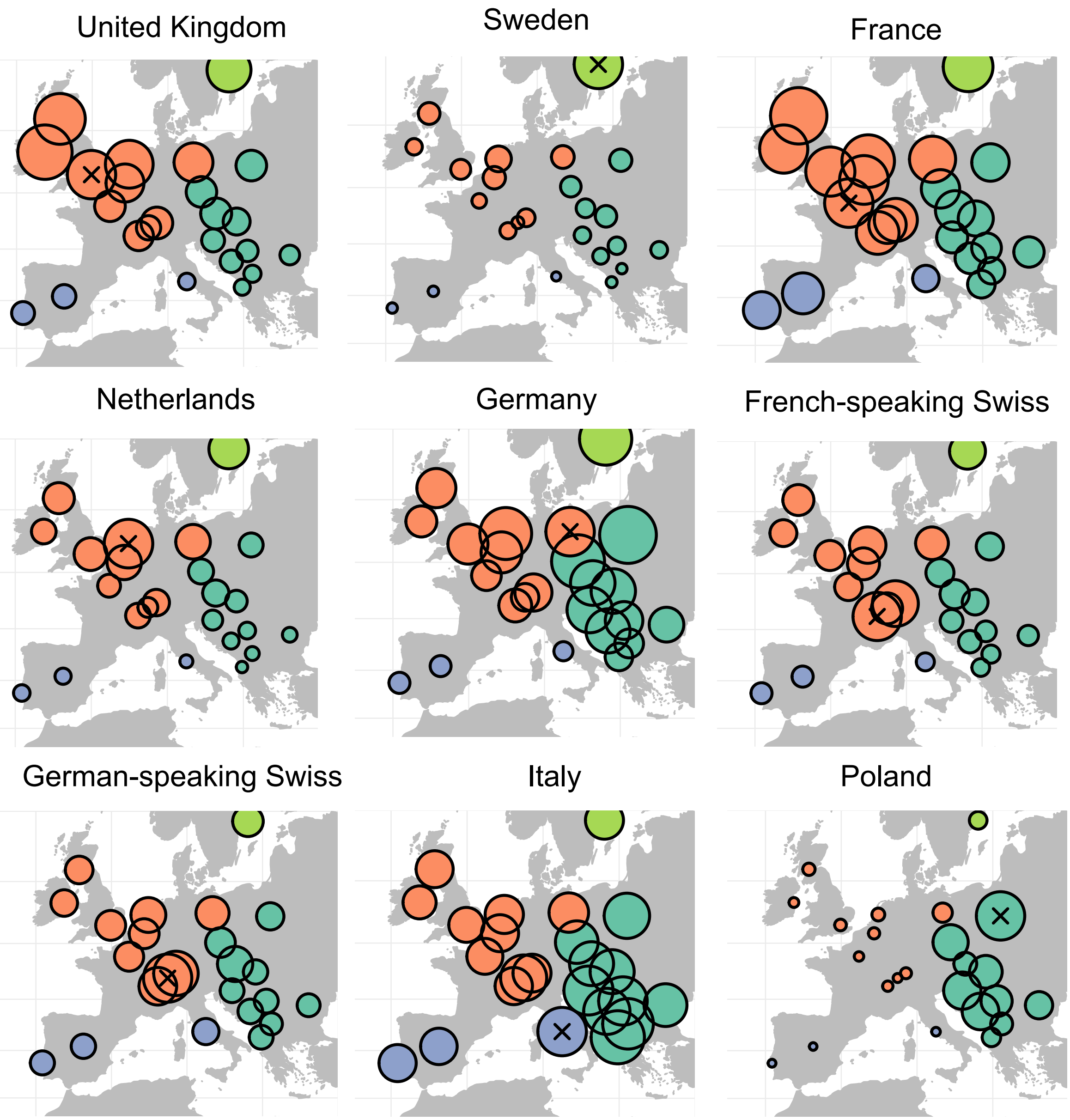
United Kingdom
Sweden
France
Netherlands
Germany
French-speaking Swiss
German-speaking Swiss
Italy
Poland
